# Plastic Shavings by Laser: Peeling Porous Graphene Springs for Multifunctional All‐Carbon Applications

**DOI:** 10.1002/advs.202301208

**Published:** 2023-05-10

**Authors:** Chanwoo Kim, Eunseung Hwang, Jinhyeong Kwon, Tae Hwan Jang, Won Chul Lee, Shi Hyeong Kim, Jongmin Park, Ming‐Tsang Lee, Hyun Kim, Sukjoon Hong, Habeom Lee

**Affiliations:** ^1^ School of Mechanical Engineering Pusan National University Busan 46241 Republic of Korea; ^2^ Optical Nanoprocessing Lab Department of Mechanical Engineering BK21 FOUR ERICA ACE Center Hanyang University Ansan Gyeonggi‐do 15588 Republic of Korea; ^3^ Intelligent Manufacturing System R&D Department Korea Institute of Industrial Technology Cheonan Chungcheongnam‐do 31056 Republic of Korea; ^4^ Division of Electrical Engineering Hanyang University ERICA Ansan Gyeonggi‐do 15588 Republic of Korea; ^5^ Department of Mechanical Engineering BK21 FOUR ERICA ACE Center Hanyang University Ansan Gyeonggi‐do 15588 Republic of Korea; ^6^ Department of Advanced Textile R&D Korea Institute of Industrial Technology Ansan Gyeonggi‐do 15588 Republic of Korea; ^7^ HYU‐KITECH Joint Department Hanyang University Seoul 04763 Republic of Korea; ^8^ Advanced Materials Division Korea Research Institute of Chemical Technology Daejeon 34114 Republic of Korea; ^9^ Department of Power Mechanical Engineering National Tsing Hua University Hsinchu 30013 Taiwan

**Keywords:** laser‐induced graphene, laser‐induced pyrolysis, pyrolytic jetting, springs, unconventional fabrication

## Abstract

Manufacturing strategies to create three‐dimensional (3D) structures with multifunctional nanomaterials are of intense interest for fabricating building blocks in many electromechanical applications. A coil spring composed of graphene provides an important step toward the realization of all‐carbon devices, as it is one of the essential elements for a wide range of systems. In this connection, here an unprecedented fabrication strategy to create a new type of 3D coil spring composed of laser‐induced graphene springs (LIG‐S) which is spontaneously produced via the pyrolytic jetting technique, is presented. Similar to wood or metal shavings observed in traditional machining processes, a pair of LIG‐S with two opposite chiralities and controllable macroscopic dimensions is produced by a single scanning of a focused continuous‐wave (CW) laser on a polyimide (PI) substrate. The resulting LIG‐S, plastic shavings by laser, exhibits sufficient mechanical and electrical properties to enable many applications including strain‐tolerant spring electrodes, antennas, supercapacitors, gas sensors, and luminescent filaments under extreme conditions. Without using any conventional fabrication techniques or other labor‐intensive, time‐consuming, and expensive processes, this novel approach enables a high‐throughput mass production of macro‐, micro‐, and nanoscale featured LIG‐S that can be manufactured within seconds to realize many open opportunities in all‐carbon electromechanical systems.

## Graphical abstract

A high‐throughput mass‐production strategy for three‐dimensional (3D) structured laser‐induced graphene springs (LIG‐S) is presented here. By a single scanning of laser on a polyimide substrate, macro‐, micro‐, and nanoscale‐featured LIG‐S can be manufactured in seconds. A number of all‐carbon electromechanical applications, including springs, antennas, supercapacitors, gas sensors, and luminescent filaments, are demonstrated.

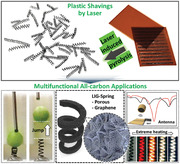

## 1 Introduction

Materials and structures are both critical as electromechanical components that are designed to function in diverse applications, including sensors,^[^ ^1^ ^]^ actuators,^[^ ^2^ ^]^ antennas,^[^ ^3^ ^]^ and flexible electronics.^[^ ^4^ ^]^ As nanoscale materials often provide novel properties compared to their bulk counterparts, strategies for designing and assembly of nanomaterials into micro‐ or macro‐structured devices are of significant interest with the aim of producing mechanically and electrically functioning components.^[^ ^1^, ^5^ ^]^ Furthermore, as the geometries of devices and parts dictate their functions, when applied to electromechanical systems, manufacturing of designed three‐dimensional (3D) structures can impart multifunctionalities beyond those achievable with two‐dimensional (2D) structures. However, high‐throughput mass production of 3D macro‐ and microstructured nanomaterials remains a challenge through conventional manufacturing, as many conventional substrates and processes are inherently 2D.^[^ ^6^ ^]^

A compelling class of nanomaterials, carbon allotropes, including low‐dimensional carbon‐based nanomaterials, exhibit remarkable material properties.^[^ ^7^ ^]^ As a consequence, these carbon allotropes, graphene^[^ ^8^ ^]^ and carbon nanotubes (CNTs),^[^ ^9^ ^]^ have been actively investigated and applied to applications on different spatial scales from ultrasmall electronic devices^[^ ^10^ ^]^ to gigantic mechanical structures,^[^ ^11^ ^]^ all with the aim of surpassing the current technological status quo. In this regard, all‐carbon devices are an attractive approach, where carbon allotropes are assembled into high‐performance devices composed of a single chemical element.^[^ ^12^ ^]^ Proof‐of‐concept demonstrations of all‐carbon devices have been focused mainly on electronic systems such as transistors,^[^ ^13^ ^]^ energy devices,^[^ ^14^ ^]^ and sensors,^[^ ^15^ ^]^ however, the all‐carbon approach can be further extended to embrace other mechanical parts to realize more sophisticated, multifunctional mechatronic devices in the future.

An arbitrary device, while on the other hand, often can be disassembled into smaller components and one of the basic building blocks for a wide range of electromechanical applications is a helical coil spring.^[^ ^16^ ^]^ The conventional 3D coil spring is compressed or stretched to store and release mechanical energy, however, an identical coil can act as an inductor if electric current flows through its terminals. Being compatible with application as both a mechanical spring and electrical inductor, a coil spring composed of conductive carbon allotropes is expected to be a crucial component for all‐carbon electromechanical devices.

At the nanoscopic scale, twisted CNTs that behave like elastic springs can be prepared from a CNT forest^[^ ^17^ ^]^ synthesized by catalytic chemical vapor deposition.^[^ ^18^ ^]^ Even though twisted CNT yarns and coils have achieved great success for actuators^[^ ^19^ ^]^ and energy harvesters,^[^ ^20^ ^]^ mass production of the CNT forest remains a concern. In addition, the complex tethering required to fix twisted CNT configurations has thus far prevented their practical deployment.^[^ ^9^, ^21^ ^]^ A more general approach compatible with different carbon allotropes is to prepare polymer nanocomposites that include carbon‐based nanomaterials as fillers. Combined with molding or 3D printing techniques, an arbitrary carbon‐based 3D architecture including a coil spring can be produced,^[^ ^22^ ^]^ however, the printed product is often lacking as an all‐carbon structure, with a relatively low loading of carbon nanofiller in general.^[^ ^23^ ^]^

A well‐known route for obtaining structured carbon allotropes is the thermochemical decomposition of organic materials.^[^ ^24^ ^]^ Among the different approaches, rapid pyrolysis of organic precursors such as polyimide (PI)^[^ ^25^ ^]^ and wood^[^ ^26^ ^]^ using lasers has received particular attention as a simple method to obtain patterned laser‐induced graphene (LIG), which has also been proven to be a compelling candidate for versatile applications.^[^ ^27^ ^]^ Conventional LIG, however, is intended for quasi‐2D graphene‐based applications as it is embedded in the mother substrate^[^ ^28^ ^]^ Despite the modification of the target substrate and the process flow further enabling the creation of free‐standing LIG strands^[^ ^15^, ^29^ ^]^ (and even arbitrary 3D LIG structures^[^ ^30^ ^]^), the production of coil springs based on the corresponding technique still remains a challenging and time‐consuming task. In this regard, we introduce a highly efficient method based on the pyrolytic jetting phenomenon^[^ ^31^ ^]^ that enables the mass production of free‐standing 3D helical LIG springs (LIG‐S) at an unprecedented speed. Instead of building a coil spring in a layer‐by‐layer manner,^[^ ^32^ ^]^ in the proposed method, LIG‐S are spontaneously extruded as a result of simple straight‐line scanning, similar to wood or metal shavings in traditional machining. The resultant LIG‐S demonstrates outstanding compatibility with untethered and multifunctional electromechanical applications, and their performance in harsh environments further enhanced their prospects for application in advanced and robust all‐carbon devices compatible with hazardous surroundings. Considering the significant recent progresses in LIG‐based sensors,^[^ ^33^ ^]^ electronics,^[^ ^34^ ^]^ and fabrication strategies for 3D structures,^[^ ^35^ ^]^ the presented high‐throughput manufacturing of LIG‐S can be further deployed as a platform technique toward a variety of multifunctional all‐carbon applications.

## 2 Results and Discussion

### 2.1 Fabrication of LIG‐S

**Figure 1:**
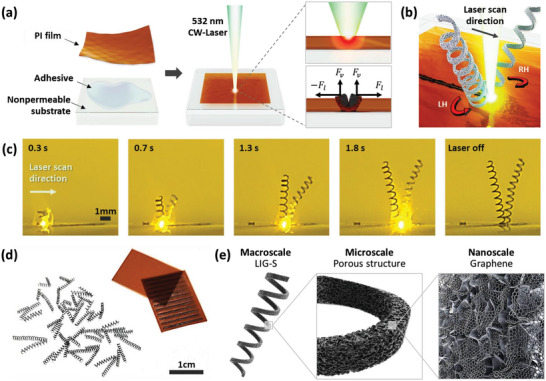
Laser‐induced graphene springs (LIG‐S) fabrication through the pyrolytic jetting process. a) Experimental procedure and configuration of the pyrolytic jetting process. b) Formation of left‐handed (LH) and right‐handed (RH) helical LIG‐S through laser scanning. c) Snapshots of LIG‐S formation during laser scanning. d) Photograph showing a number of LIG‐S manufactured from a single polyimide (PI) film in less than 1 min. e) Schematic illustration of a resultant LIG‐S at macro‐, micro‐, and nanoscale.

The proposed manufacturing procedure of the LIG‐S was discovered through observation of the plastic shavings produced during the pyrolytic jetting process^[^ ^31^ ^]^ under a carefully designed experimental configuration, as shown in **Figure** **1**a. Using the conventional pyrolytic jetting process, it was verified that once a highly focused continuous‐wave (CW) laser is rapidly scanned over a PI matrix, the intensive pressure created by the pyrolysis gas at the scanning front induces spontaneous exfoliation of the highly expanded LIG in the direction opposite to the scanning path. In this study, we found that the product from the pyrolytic jetting process can be transformed from simple LIGs into LIG‐S when two additional conditions are satisfied: 1) the thickness of the PI film is sufficiently thin, and 2) the corresponding PI film is in conformal contact with the underlying nonpermeable substrate by capillary adhesion.^[^ ^36^ ^]^ Owing to the Gaussian intensity profile of the focused laser followed by the subsequent light absorption and heat transfer within the substrate, when the PI film is sufficiently thin (≈75 µm), the heat‐affected zone created by the scanning laser can be approximated as a one‐dimensional (1D) profile with a higher temperature region at the center. In a typical conversion of PI into LIG, the tensile strength of the resultant LIG tends to decrease as the laser power increases, owing to the higher degree of graphitization.^[^ ^37^ ^]^ As a consequence, the central region of the scanning path becomes more susceptible to pressure‐induced rupture when accompanied by a higher pressure at the corresponding spot. As the LIG undergoes separation, the inner pressure acts as a centripetal force that results in helical jetting of the exfoliated LIG when combined with the original jetting trajectory. Note that, in these experiments, very few LIG‐S were generated in the absence of either condition, as explained in more detail in Figure S1 (Supporting Information). Because the lateral forces are exerted in opposite directions (F*_l_* and −F*_l_*) on the LIGs, a single scan results in the simultaneous creation of a pair of LIG‐S having different chiralities, as illustrated in Figure 1b. This occurs unless an intentional asymmetry is introduced to the experimental configuration, for example, through beam shaping or by deliberate tilting of the sample stage under scanning, as shown in Figure S2 (Supporting Information). The snapshots in Figure 1c were captured from real‐time video footage captured during the manufacturing process (Movie S1, Supporting Information). This confirms that a pair of right‐handed (RH) and left‐handed (LH) helices were jetted simultaneously toward the two perpendicular directions of the scanning path at an unprecedented rate, being fully formed within just 2 s. The proposed fast and simple laser scanning fabrication method enables mass production of 3D porous LIG‐S, with numerous LIG‐S produced from a single piece of PI film within <1 min, as shown in Figure 1d. The macro‐, micro‐, and nanoscale morphological characteristics of a resulting LIG‐S are illustrated in Figure 1e.

### 2.2 Processing Parameters for LIG‐S Geometry Controls

**Figure 2:**
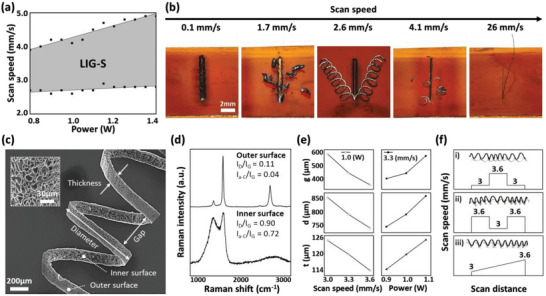
Processing parameters and laser‐induced graphene springs (LIG‐S) geometry control. a) Laser scanning speed versus laser power plot indicating acceptable LIG‐S manufacturing conditions. b) Optical images showing the results of varying laser scanning speed (0.1–26 mm s^−1^) at a fixed power (1.0 W) and scan length (6 mm). c) Scanning electron micrograph (SEM) of a sample LIG‐S and nomenclature of geometric parameters; inset shows a high‐magnification SEM image demonstrating the porous structure of the LIG‐S. d) Results of Raman spectroscopy for the outer and inner regions of sample LIG‐S. e) LIG‐S geometry control according to laser scan speed and power. f) In situ LIG‐S geometry control through the adjustment of the laser scan speed during a single scanning process.

The laser‐processing parameters required for LIG‐S manufacturing were also investigated. The combinatorial study of laser power versus scanning speed in **Figure** **2**a,b shows that there exists a large processing window for the generation of LIG‐S, which is beneficial from the perspective of future scalability. The morphology of the LIG‐S is analogous to the LIG reported elsewhere, consisting of highly porous carbon nanostructures, as shown in the high‐magnification scanning electron micrograph (SEM) images in Figure 2c, because of the large volume expansion that occurs during pyrolytic jetting.^[^ ^31^ ^]^ Meanwhile, considering the jetting trajectory, the outer and inner surfaces of the helical structure experience different thermal histories, leading to spatially distinguishable material compositions that also result in diverse physical properties. Figure 2d shows the representative Raman spectra obtained from the two regions of a LIG‐S. The outer surface, subjected to the higher laser intensity, presents a higher degree of conversion into LIG, as confirmed by the clear graphene (G) peak without considerable signs of defects (D) as well as the low presence of amorphous carbon (a‐C) compositions. On the other hand, the characteristics of the inner surface are uneven compared to the outer surface owing to the broadened temperature gradient at the edge, which yields more irregular jetting boundaries.^[^ ^38^ ^]^ Together with the morphological characteristics, the *I*_D_/*I*_G_ and *I*_a‐C_/*I*_G_ ratios, respectively, increased to 0.90 and 0.72, at the inner surface, from 0.11 and 0.04 at the outer surface. Thus, the broadening of the characteristic peaks indicated that the inner surface contained a significant proportion of a‐C molecules.^[^ ^39^ ^]^ The difference in material properties between the inner and outer surfaces may facilitate additional functionalities in the LIG‐S, analogous to the bilayer spring consisting of layers with dissimilar responsivity to external stimulation for adaptive locomotion of microrobots.^[^ ^40^ ^]^ More detailed data measured from different spots of identical LIG‐S are included in Figure S3 (Supporting Information).

From a macroscopic perspective, a shift in the laser parameters results in the alteration of the LIG‐S geometries, as shown in Figure 2e, which is also related to the performance of the mechanical coil spring.^[^ ^41^ ^]^ In the optical configuration used in this study, the variable dimensional parameters of a LIG‐S include its diameter (d), gap (g), and thickness (t), which can be tuned by controlling the laser scanning speed, power, and focus distance (Figure S4, Supporting Information). The mechanical and electrical properties of the coil spring are controllable by simple adjustment in the laser condition, however, we predict possibilities for further extension of these dimension ranges if the other fixed experimental parameters, such as the thickness of the PI film (Figure S5, Supporting Information), laser wavelength, and the size of the focused beam spot, can be also varied.

The LIG‐S investigated further in this study were all produced under similar conditions, yielding LIG‐S with a spring index of 6–7. Adjustment of the laser parameter in situ during exfoliation, on the other hand, resulted in a coil spring with dimensions varying in both discrete (Figure 2f, i and ii) and continuous (iii) manners. Such immediate reflection of the laser irradiation condition permits the creation of nonlinear variable‐rate springs that provide an unconventional strain–force response.^[^ ^42^ ^]^ We predict that more complex structures can also be jetted by an identical approach once coupled with active control of the processing parameters. Based on this mass production capability and geometrical controllability, we further expect even better high‐throughput manufacturing potential if other optical schemes, such as microlens arrays with multiple beamlets,^[^ ^43^ ^]^ are incorporated.

### 2.3 Mechanical and Electrical Properties of LIG‐S

**Figure 3:**
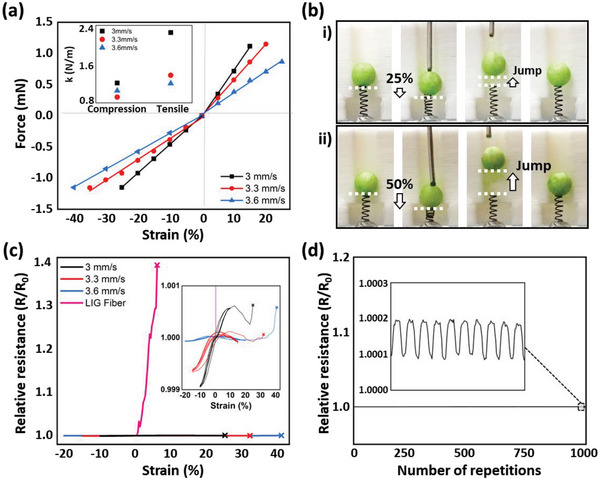
Laser‐induced graphene springs (LIG‐S) mechanical and electrical characteristics. a) Force–strain curve of three geometrical types of LIG‐S obtained from different laser scan speeds (3–3.6 mm s^−1^). b) Snapshots of jumping Styrofoam ball actuated by the elastic recovery of a LIG‐S. c) Electrical resistance changes during the mechanical deformation of a LIG‐S and LIG‐fiber. d) Relative resistance of a LIG‐S during 1000 cycles of 25% strain.

The main objective of a conventional helical spring is to store mechanical energy and exert a force that is ideally proportional to its displacement from its relaxed length, according to Hooke's law. The force–strain curve for a single LIG‐S was measured and plotted in **Figure** **3**a. The LIG‐S spring constants, determined from the slope of each curve, were in the range of 0.8–2.4 N m^−1^ under the conditions of this study, and these values were several orders of magnitude higher than those of the graphene fiber springs reported previously.^[^ ^44^ ^]^ The equation for the elasticity coefficient of a helical spring,^[^ ^44^ ^]^ although the detailed geometry of the spring is not completely matched, estimates that the shear modulus of the resulting LIG‐S is ≈0.3 GPa (Summary of electrical and mechanical properties concerned with free‐standing LIGs is described in the Table S1, Supporting Information). This is considerably lower than the shear modulus of fully dense isotropic graphite^[^ ^45^ ^]^ or other carbon products from pyrolysis,^[^ ^46^ ^]^ but comparable to that measured from LIG converted from a PI substrate^[^ ^37^ ^]^ when the effect of volume fraction porosity on the shear modulus^[^ ^45^ ^]^ is reflected. At the same time, the minor suppression of the spring constant in the direction of compression compared to the tensile case, as shown in the inset, appears to be associated with the porosity of the specific LIG‐S.^[^ ^47^ ^]^ The high spring constant of the LIG‐S manufactured using the proposed method enables the storage of elastic energy at a macroscopic level, which has been challenging thus far, owing to springs with a low coefficient of elasticity^[^ ^48^ ^]^ presenting an insignificant force–strain curve under a compression–release cycle due to the effects of their own weight. A simple demonstration of the LIG‐S as a mechanical spring (Figure 3b) confirms that the potential energy stored in the LIG‐S is converted into the kinetic energy of a Styrofoam ball, increasing its height against the effect of gravity. In this experiment, the maximum height achieved at (ii) 50% compression was significantly higher than that at (i) 25% strain because the energy released by the LIG‐S is proportional to the square of its linear deformation.

On the other hand, the electrical conductivity of LIG‐S shows a negligible change according to the applied strain, as shown in Figure 3c, for all samples created under different scanning conditions in this study. The magnified graph in the inset confirms that the electrical resistance change within the accepted strain range is less than ±0.1%, implying that the resulting LIG‐S is suitable for wiring in stretchable devices.^[^ ^49^ ^]^ The advantage of LIG‐S becomes more evident when compared to the strain‐dependent electrical resistance of a conventional LIG fiber, which displays a rapid increase in resistance with increasing applied strain and fails at a strain of ≈5%. Note that the strain‐tolerant electrical conductivity of LIG‐S remained stable, as confirmed from the cyclic stretching test performed 1000 times (Figure 3d). Meanwhile, the maximum strain applicable to the LIG‐S produced by the proposed method was ≈40%, as denoted in the plot. However, we would like to stress that LIG‐S with a higher strain capability can also be created with the proposed technique (Figure S6, Supporting Information).

### 2.4 Applications of LIG‐S

**Figure 4:**
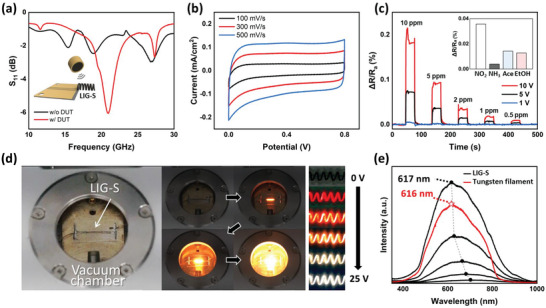
Applications based on the unique structural and material characteristics of the laser‐induced graphene springs (LIG‐S). a) Antenna: S‐parameter (S_11_) of the LIG‐S antenna showing the operating frequency band. b) Supercapacitor: Cyclic voltammetry (CV) curves of the LIG‐S at various voltage scan rates. c) Chemoresistive gas sensor: Detection capabilities of the LIG‐S for NO_2_ gas at various input voltages; inset shows the reactivity of the LIG‐S gas sensor to various chemicals. d) Luminescent filament: Characteristics of the resistively heated LIG‐S in a vacuum chamber. e) Spectrum of emitted light from LIG‐S and commercial tungsten filament during resistive heating.

While carbon is a universal and versatile element, as discussed above, LIG‐S has the multiscale structure of a hierarchical spring comprising highly porous LIG and other carbon allotropes. These characteristics at the macro‐, micro‐, and nanoscale are vital for various applications, including wireless communication, energy storage, and environmental sensing, which are all enabled by equivalent LIG‐S harnessing the respective features from each scale. For example, the evident coil‐shaped conductive structure of LIG‐S enables their feasible utilization as helical antennas.^[^ ^50^ ^]^ A helical antenna consists of one or more conducting wires wound in the form of a helix. **Figure** **4**a shows the S‐parameter of the proposed LIG‐S helical antenna device under test (DUT), measured by a vector network analyzer, with the measurement setup shown in Figure S7 (Supporting Information). The S‐parameter indicates the antennas’ reflected voltage against the incident voltage; the smaller the dB value, the smaller the return loss.^[^ ^51^ ^]^ The S‐parameter measured with and without the DUT was −6 and −1.49 dB, respectively, at a frequency of 20.95 GHz. This means that the radiation of the input power was increased from 18% to 75% by adding the DUT. These results indicate that the LIG‐S antenna is a potential candidate antenna in the millimeter wave band, such as in the 5 G frequency range.

On the other hand, the highly porous LIG‐S morphology provides a high surface area‐to‐volume ratio, which is crucial to achieving a higher energy storage density for energy devices.^[^ ^52^ ^]^ Cyclic voltammetry (CV) measurements were taken to evaluate the electrochemical performance of a sample LIG‐S as an electrical double‐layer capacitor in 1 m Na_2_SO_4_ aqueous solution. As shown in Figure 4b, the obtained CV curves exhibit a quasi‐rectangular profile, suggesting the ideal capacitive behavior of the LIG‐S electrode. Moreover, the performance of the LIG‐S was evaluated based on its galvanostatic charge–discharge (GCD) curves with the same electrolytes, as presented in Figure S8 (Supporting Information). The specific capacitance of the LIG‐S obtained from the GCD at different input current densities exhibited a gradual decrease from 1.59 mF cm^−2^ at 1.42 µA cm^−2^ to 0.37 mF cm^−2^ at 8.55 µA cm^−2^. This reveals that even without any electrochemical optimization process, the energy storage performance of the LIG‐S is comparable to that of graphitized nanoscale porous carbon produced by a high‐resolution laser.^[^ ^33^, ^53^ ^]^ Also, the LIG‐S retains its electrochemical performance despite repeated deformation or changes in temperature due to its intrinsic structural and material properties (Figure S9, Supporting Information).

Apart from these structural characteristics, graphene and other allotropic carbons are known to be responsive to a wide range of chemical compounds,^[^ ^54^ ^]^ as are LIG‐S under volatile gases in their surroundings. Figure 4c shows the magnitude of the electrical response in a sample LIG‐S in terms of the normalized electrical resistance change measured at different NO_2_ gas concentrations (see Figure S10, Supporting Information for the detailed experimental setup). The working mechanism of carbon nanomaterial‐based chemoresistive gas sensors is usually presumed to be the surface adsorption of target gaseous species that shifts the Fermi level of the conjugated carbon nanomaterial,^[^ ^54^ ^]^ but additional mechanisms, such as the effect of the surface water layer^[^ ^55^ ^]^ may contribute to the overall performance. The exact understanding of LIG‐S as a chemoresistive gas sensor is therefore challenging, yet its response in the above test indicates that the LIG‐S gas sensor can distinguish different concentrations of NO_2_. However, its sensitivity to other volatile compounds was measured to be relatively small compared to NO_2_, as summarized in the inset, which is similar to other carbon nanomaterial‐based gas sensors reported previously.^[^ ^56^ ^]^ Furthermore, the unique properties of LIG‐S, which can respond to various stimuli such as different gases, mechanical deformation, and temperature changes, make it a promising candidate for the development of multimodal sensors. However, to utilize LIG‐S as a single sensing element for detecting multiple input signals, a separate decoupling mechanism is required, which is a significant challenge. Several studies have reported on the development of decoupling mechanisms for multimodal sensors.^[^ ^33a^ ^]^ The high productivity of LIG‐S can be advantageous for the integration of multiple LIG‐S sensing elements on a single platform, enabling the development of multimodal sensors. Moreover, the development of functionalized LIG specific to sensing targets^[^ ^33d^ ^]^ allows the fabrication of arrays of functionalized LIG‐S sensors, which can detect various stimuli individually. With the growing interest in data‐driven approaches,^[^ ^57^ ^]^ including deep learning, there is great potential for further advancements in LIG‐S‐based multimodal sensors.

According to our research, the three applications presented above are crucial elements for untethered devices and could, for example, be applied in an isolated environmental detector that collects information about the ambient environment and sends it back to distant users. Furthermore, as LIG is known to be chemically stable,^[^ ^58^ ^]^ it is anticipated that the proposed LIG‐S can withstand harsh environments, such as high temperatures. This is supported by the fact that carbon filaments have long been used for incandescent bulbs.^[^ ^59^ ^]^ Figure 4d presents photographs of a LIG‐S that underwent stepwise resistive heating inside a vacuum chamber (see Figure S11, Supporting Information, for the detailed experimental setup). Increasing the magnitude of the applied voltage resulted in an increase in the luminescence, as is evident from the images, while magnified images confirm simultaneous alteration in the overall color of the emitted light. The spectrum captured through the observation window (Figure 4e) quantitatively validates that the peak wavelength of the emitted light experienced a blue shift as the amount of resistive heating increased. The spectrum emitted from the LIG‐S became comparable to that of a commercial tungsten filament, which generally operates at an extremely high temperature of ≈3000 K.^[^ ^60^ ^]^

## 3 Conclusion

In conclusion, this study demonstrates that the pyrolytic jetting technique can be effectively applied for the high‐throughput mass production of helical springs composed of hierarchical LIG with a highly porous morphology, which has great potential as a core element for all‐carbon electromechanical devices. By varying the laser conditions, the detailed parameters of the resulting LIG‐S, including its geometrical dimensions and material compositions, were controlled, which in turn adjusted the mechanical and electrical properties of the resultant LIG‐S to produce a customized helical spring on demand. Immediate reflection of the changes in the laser parameters during the process further substantiates that the corresponding technique is not only applicable to helical springs, but also potentially expandable to arbitrary shapes in the future.

The resultant LIG‐S operates as a conventional mechanical spring and strain‐tolerant stretchable electrode, however, the structural features of LIG‐S enable additional applications that are useful for all‐carbon untethered devices once coupled with the unique properties of the constituent carbon‐based nanomaterials. We would like to emphasize that LIG‐S‐based helical antennas, supercapacitors, and gas detectors, which are selected as representative examples from wireless communication, energy storage, and environmental sensing applications, are all demonstrated on comparable LIG‐S produced under a single laser condition. It is therefore anticipated that there is significant potential for improvement in terms of specific device performance for each application through optimization of the jetting parameters or modification of the process itself, for example, precoating of a highly conductive layer on the mother substrate before jetting to enhance the capacitance of energy devices.^[^ ^61^ ^]^

Because carbon allotropes have versatile usages,^[^ ^62^ ^]^ tasks available by LIG‐S are not limited to those demonstrated in this study. Among others, we expect that the energy‐ and environment‐related applications of carbon nanomaterials, such as in photocatalytic water purification^[^ ^63^ ^]^ and electrochemical energy harvesting,^[^ ^64^ ^]^ are closely related to the current discussion of using LIG‐S in degraded environments. Meanwhile, the helical shape is also potentially advantageous for application as the main frame of microrobots that undergo programmed locomotion according to external signals.^[^ ^65^ ^]^ In combination with the mass productivity of the proposed production technique and the exceptional tolerance to harsh environments of the resulting LIG‐S, we anticipate that the LIG‐S can be developed as a basic unit for a swarm of untethered microrobots^[^ ^66^ ^]^ intended to remotely investigate, monitor, and decontaminate unknown hazardous environments.

## 4 Experimental Section

### Materials and Substrate Preparation

For conformal contact, an appropriate amount of ethylene glycol (EG) was placed as an adhesive layer onto the surface of the slide glass and then smoothly covered by a 70 µm‐thick PI film (SKC, South Korea). The EG was evenly distributed by the capillary force and used to prevent unexpected dissipation of capillary adhesion caused by evaporation.^[^ ^67^ ^]^

### LIG‐S Fabrication

A computer‐assisted laser scanning system comprising a galvano‐mirror (hurrySCAN II 14, Scanlab, Germany) and 532 nm CW laser (3 W, Laser Glow Technologies, Germany) was used for the pyrolytic jetting process. To meet LIG‐S fabrication conditions, applied laser power was controlled from 0.8 to 1.4 W at the optimal scanning speed between 2.6 and 4.9 mm s^−1^.

### Optical, Mechanical, and Compositional Characterization

SEM (ZEISS SUPRA25, Germany) was used to study the surface morphology and microstructure of the LIG‐S. A LabRAM ARAMIS Raman spectrometer was used to analyze the surface chemistry of the LIG‐S. To measure the force–strain performance, LIG‐S were connected between the upper and lower holders of a mechanical testing machine (CellScale, UniVert), where the ramp rate was fixed at 2.4 mm min^−1^. The forces during stretching and compression were measured using a connected load cell (FuTek, 1N).

### Antenna Characterization

To measure the S‐parameters, a sample LIG‐S was attached to the antenna under test after calibrating the vector network analyzer (VNA, Rohde and Schwarz ZVA50) using thru‐reflect‐line calibration. The S‐parameter, which is the ratio of the reflected wave to the incident wave, is a commonly used parameter in measuring microwave devices. During the VNA setting, the intermediate‐frequency bandwidth was set to 100 Hz, and the frequency sweep was conducted from 10 to 30 GHz.

### Electrochemical Characterization

A potentiostat (Versa STAT 3, Berwyn, PA, USA) was used to measure the CV and galvanostatic charge‐discharge behavior of the LIG‐S. LIG‐S was fabricated using laser scan conditions of 1.0 W and 3.3 mm s^−1^ for electrochemical analysis.

### Gas Sensing Characterization

Gas‐sensing performance tests were carried out using a gas sensor measurement system (GMC 1200, ATOVAC, South Korea) with data acquisition instruments (2450 Sourcemeter, Keithley, USA). The resistance of the sensor was calculated from the current recorded at the applied constant DC voltages of 1, 5, and 10 V. The gas concentration was controlled by changing the mixing ratio of nitrogen and NO_2_ (20 ppm in nitrogen) with a fixed oxygen concentration of 21%, using a mass flow controller (Model 5850E, Brooks Instrument, USA) between 0.5 and 10 ppm. Nitrogen and oxygen were used as the carrier gases at a fixed flow rate of 500 sccm. Various analyte gases (NH_3_, CH_3_COCH_3_, and C_2_H_5_OH) were used to observe the gas selectivity of the sensor at 5 ppm and 5 V. The response was used to characterize the sensor performance using the equation: Response = Δ*R*/*R*a, where Δ*R* is |Ra – Rg| and Ra and Rg are the electrical resistance of the sensor under dry air and the analyte gas, respectively.

### Luminescent Filament Test

During the resistive heating of the LIG‐S, the light intensity was measured using a spectrometer (Flame, Ocean Insight, USA). Joule heating was carried out in a vacuum chamber, and the input voltage was controlled using a source meter (2450 Sourcemeter, Keithley).

## Conflict of Interest

H.L., S.H., H.K., C.K., and E.H. are the inventors of provisional Korea patent application (No. 10‐2023‐0045545) submitted jointly by Pusan National University, Hanyang University, and Korea Research Institute of Chemical Technology, which covers the concept, fabrication, performance, and applications of LIG‐S.

## Supporting information

Supporting Information

Supplemental Movie 1

## Data Availability

The data that support the findings of this study are available from the corresponding author upon reasonable request.
