# Supplementary material for: Plastic Shavings by Laser: Peeling Porous Graphene Springs for Multifunctional All‐Carbon Applications
Source: Adv Sci (Weinh). 2023 May 10;10(21):2301208. doi: 10.1002/advs.202301208 (PMC10375133; doi:10.1002/advs.202301208)
Supplement: Supplementary file 1 — Supporting Information [file ADVS-10-2301208-s002.pdf]

ADVANCED SCIENCE
Open Access

Supporting Information

for *Adv. Sci.*, DOI 10.1002/advs.202301208

Plastic Shavings by Laser: Peeling Porous Graphene Springs for Multifunctional All-Carbon Applications

Chanwoo Kim, Eunseung Hwang, Jinhyeong Kwon, Tae Hwan Jang, Won Chul Lee, Shi Hyeong Kim, Jongmin Park, Ming-Tsang Lee, Hyun Kim, Sukjoon Hong* and Habeom Lee**

Supporting Information

Plastic Shavings by Laser: Peeling Porous Graphene Springs for Multifunctional All-carbon Applications

Chanwoo Kim[1]†, Eunseung Hwang[2]†, Jinhyeong Kwon[3], Tae Hwan Jang[4], Won Chul Lee[5], Shi Hyeong Kim[6,7], Jongmin Park[8], Ming-Tsang Lee[9], Hyun Kim[8], Sukjoon Hong[2]*, Habeom Lee[1]**

[1] School of Mechanical Engineering, Pusan National University, Busan 46241, Republic of Korea

[2] Optical Nanoprocessing Lab, Department of Mechanical Engineering, BK21 FOUR ERICA, ACE Center, Hanyang University, Ansan, Gyeonggi-do 15588, Republic of Korea

[3] Intelligent Manufacturing System R&D Department, Korea Institute of Industrial Technology, Cheonan, Chungcheongnam-do 31056, Republic of Korea

[4] Division of Electrical Engineering, Hanyang University, Ansan, Gyeonggi-do 15588, Republic of Korea

[5] Department of Mechanical Engineering, BK21 FOUR ERICA, ACE Center, Hanyang University, Ansan, Gyeonggi-do 15588, Republic of Korea

[6] Department of Advanced Textile R&D, Korea Institute of Industrial Technology, Ansan, Gyeonggi-do 15588, Republic of Korea

[7] HYU-KITECH Joint Department, Hanyang University, Seoul 04763, Republic of Korea

[8] Advanced Materials Division, Korea Research Institute of Chemical Technology, Daejeon 34114, Republic of Korea

[9] Department of Power Mechanical Engineering, National Tsing Hua University, Hsinchu 30013, Taiwan

† Contributed equally.

* *Corresponding authors:* hyunkim@krict.re.kr (Dr. H. Kim), sukjoonhong@hanyang.ac.kr (Prof. S. Hong), and hblee@pusan.ac.kr (Prof. H. Lee).

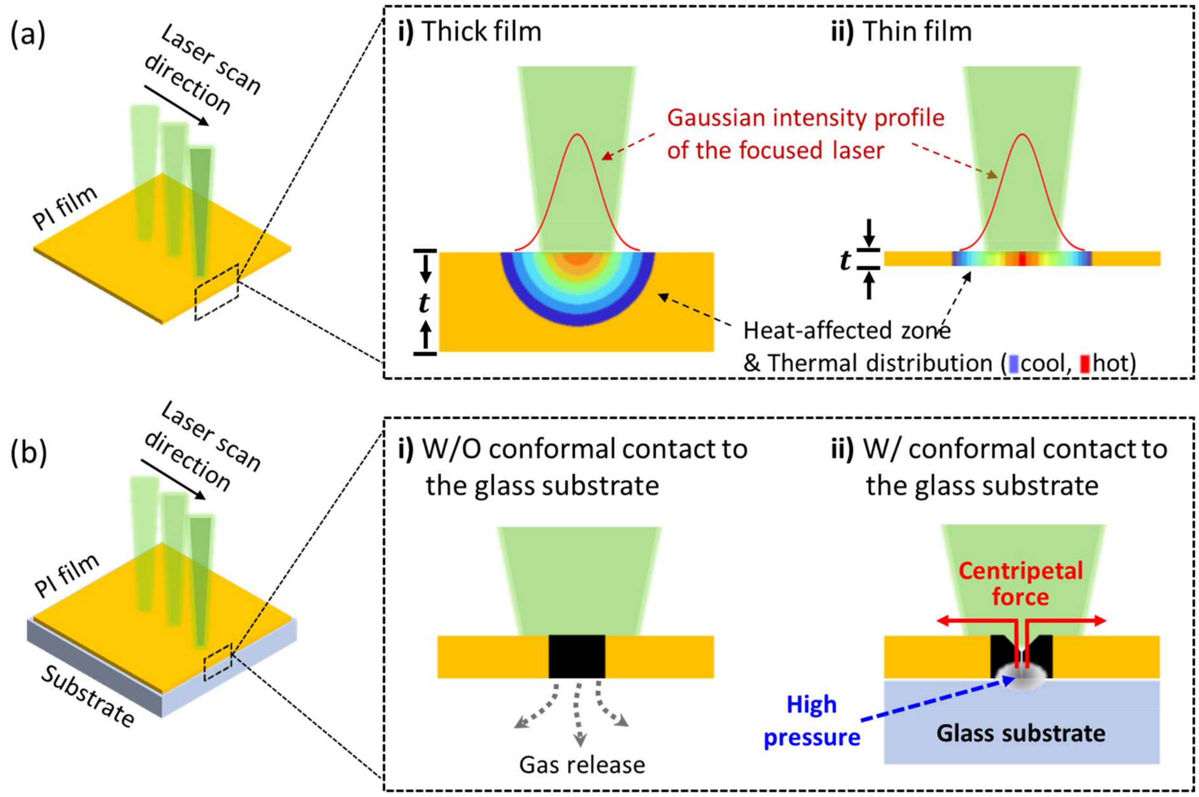

Figure S1. Illustration of the two conditions required for the transition from LIG-fiber to LIG-S. (a) Thickness of the PI film; when the thickness of the PI film is sufficiently thin, the temperature profile can be considered 1D. (b) Conformal contact between the PI film and an underlying nonpermeable substrate: the gas trapped inside increases the internal pressure, generating a centripetal force to separate the LIG-F into two parts during the laser-induced jetting process.

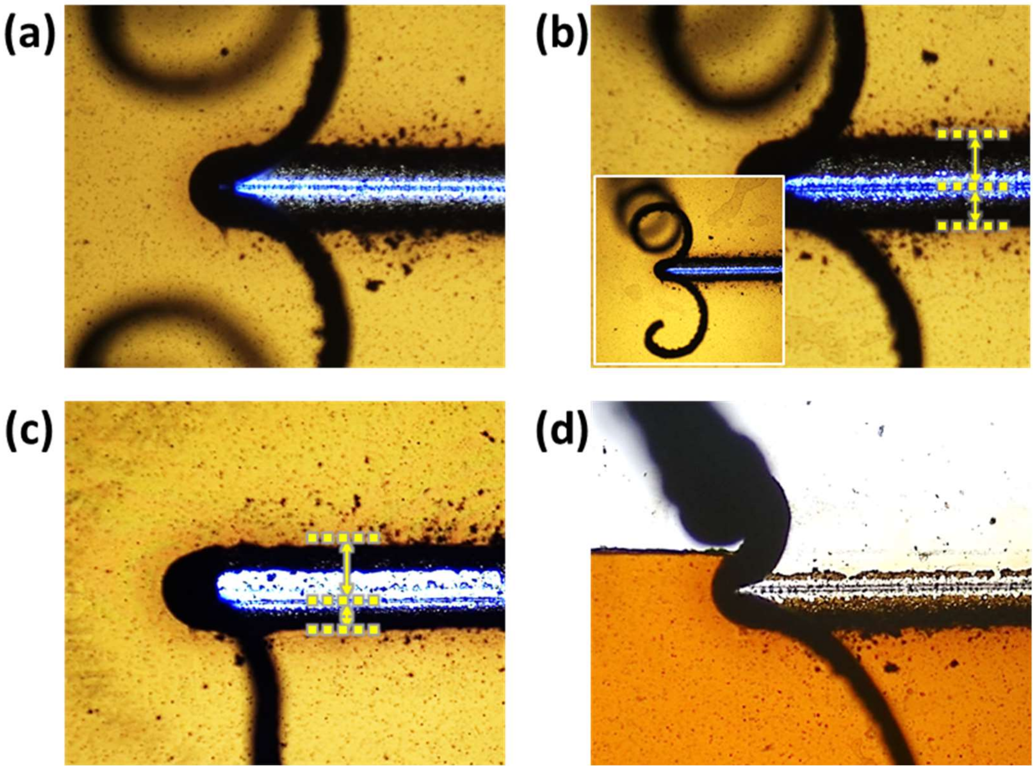

Figure S2. (a) A pair of LIG-S achieved through balanced laser scanning. The results of the intended asymmetric thermal effect by ((b) and (c)) tilting the PI films during laser scanning or (d) laser scanning through the edge of the PI film.

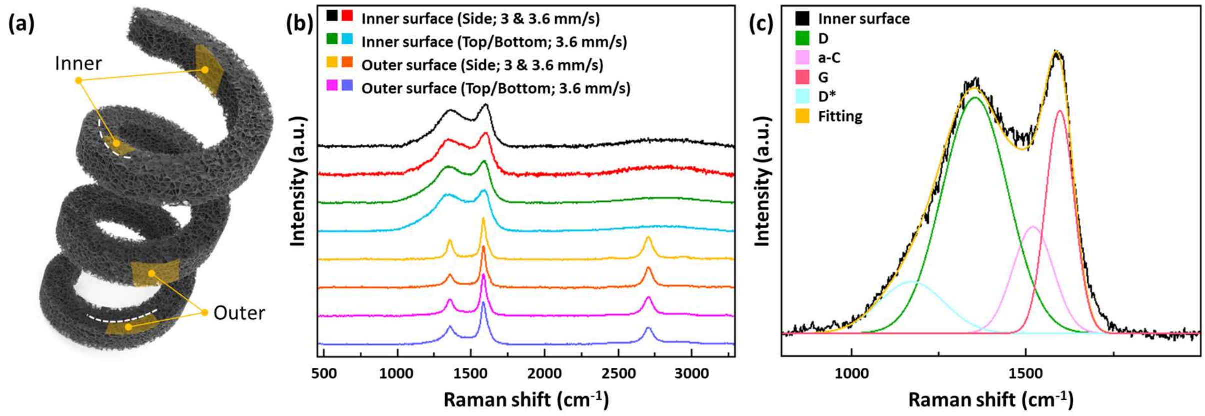

Figure S3. (a) Schematic illustration of LIG-S indicating the outer (side plane: outside the structure; top/bottom plane: outside the diameter) and inner (side plane: inside the structure; top/bottom plane: inside the diameter) regions. (b) Raman spectra of different outer and inner spots of an identical LIG-S from the side, top, and bottom planes. (c) Deconvolution of the Raman spectrum of the inner surface, including defects and amorphous carbon peaks.

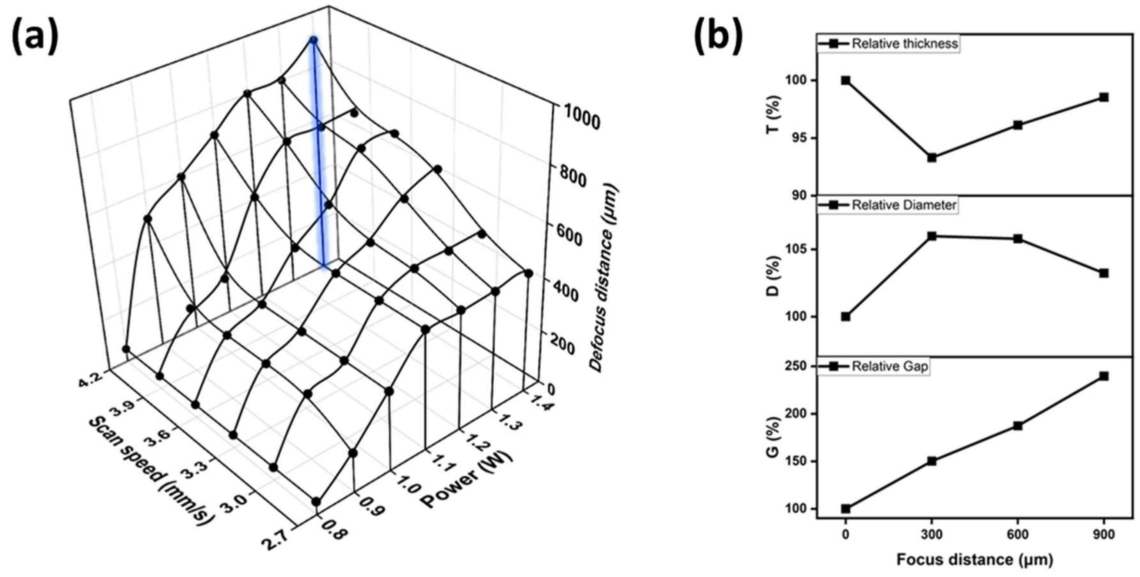

Figure S4. Plot of laser scan speed and power showing the LIG-S fabrication conditions for different focus distances. (b) Summary of the shape change of LIG-S fabricated from a common scan speed and power condition (1.4W –3.6 mm/s) with various focus distances. The blue line in (a) indicates the related fabrication conditions.

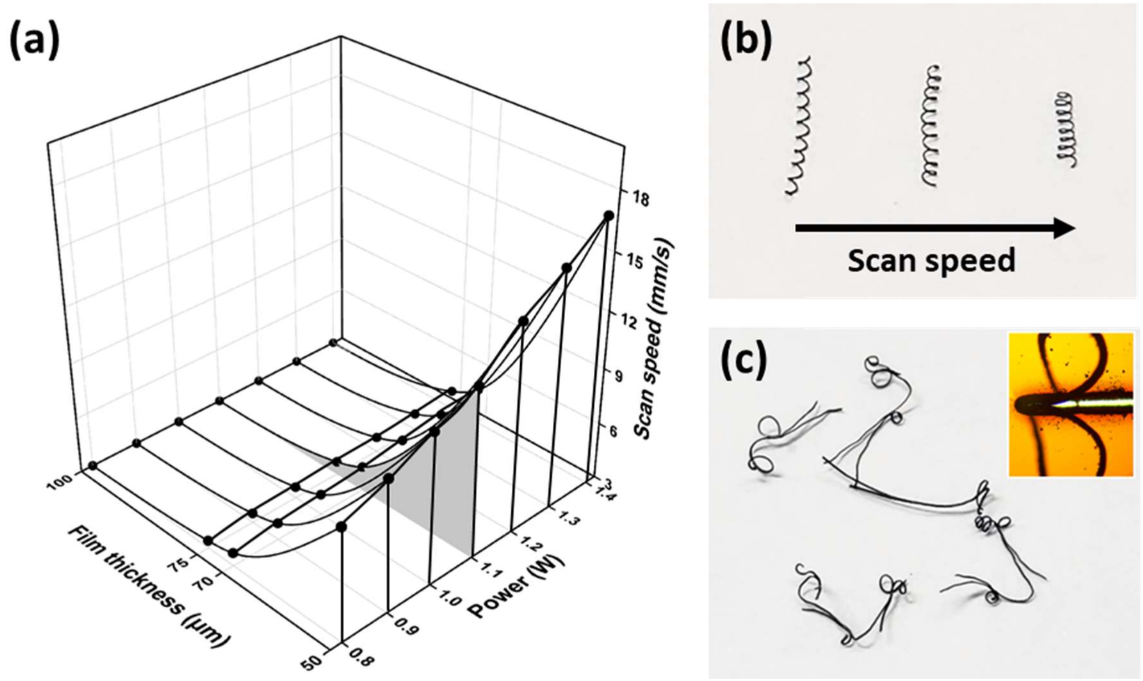

Figure S5. (a) Plot of laser scan speed and laser power showing the LIG-S fabrication conditions for different PI film thickness. The gray area indicates the variation of the effective scan speed range depending on the film thickness at a fixed laser power of 1.1 W (b) LIG-S fabricated from a 50 μm-thick PI film with scan speeds of 4, 8, and 12 mm/s. (c) LIG fibers fabricated from a PI film with a thickness of 100 μm; at 100 μm thickness, the temperature profile is hard to be regarded as 1D, and the resulting centripetal forces and inner pressure cannot induce the helical jetting of the exfoliated LIG.

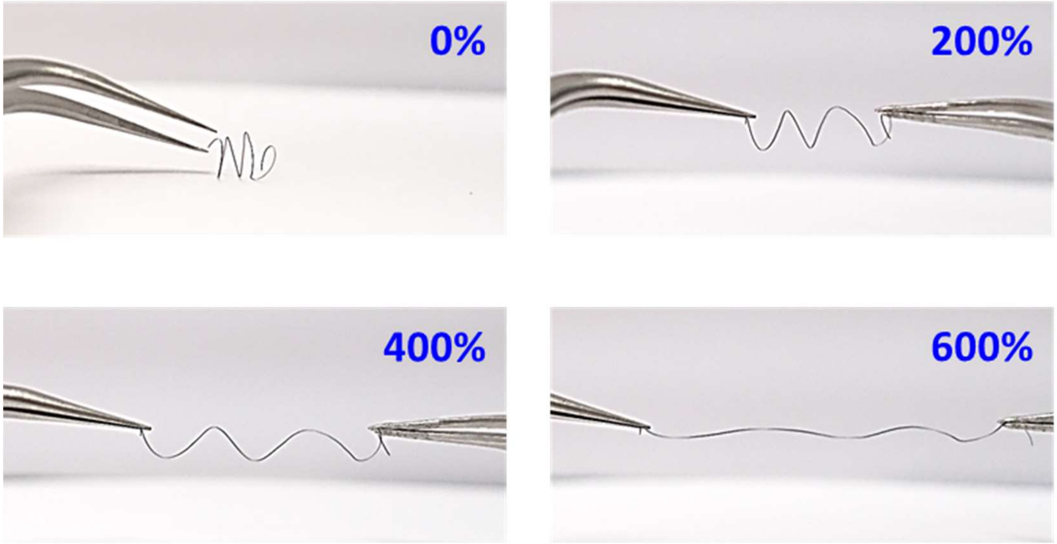

Figure S6. Digital images of LIG-S exhibiting a high stretchability of up to 600 % strain.

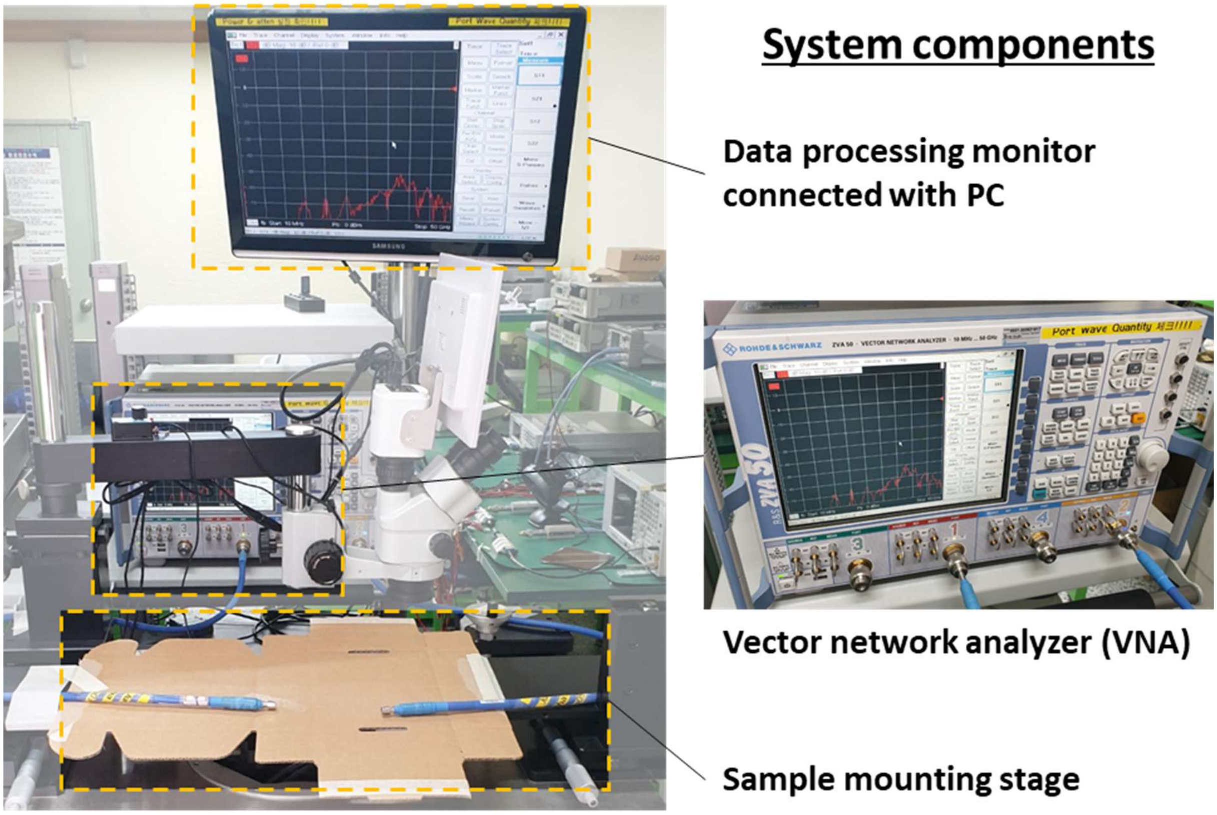

Figure S7. Digital images of probe station for S-parameter measurements.

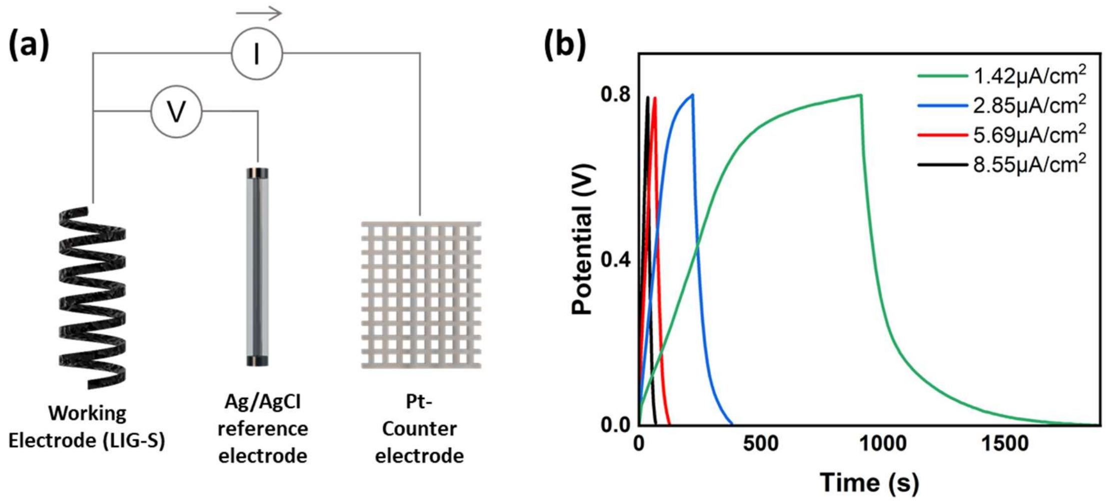

Figure S8. (a) Illustration of the setup for LIG-S CV and GCD measurements. (b) LIG-S charge–discharge behavior at various current densities.

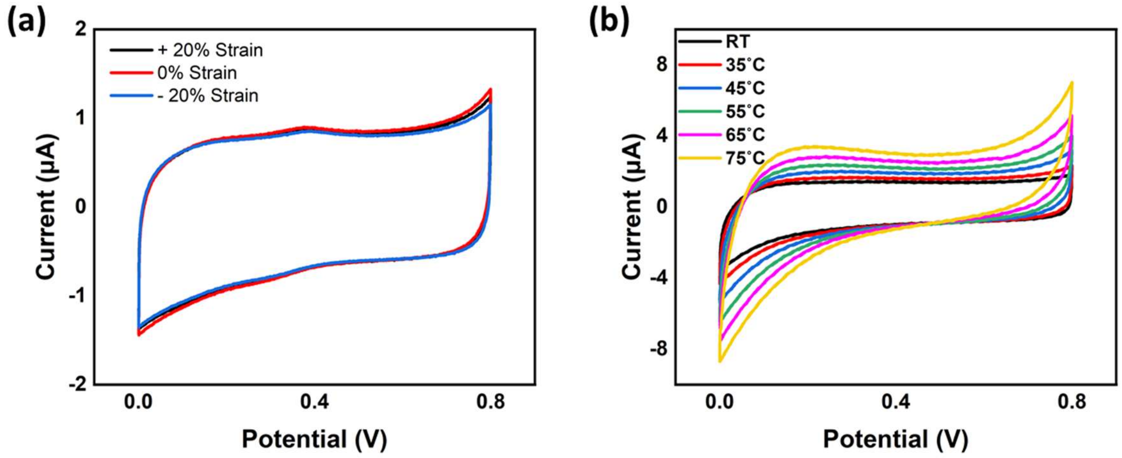

Figure S9. (a) CV graph of the LIG-S under mechanical deformations at 100 mV/s scan rate. (b) CV graph of the LIG-S under temperature changes in an aqueous Na_2SO_4 solution at 100 mV/s scan rate.; the capacitance of LIG-S increased with temperature due to the activation of LIG-S pores by improved wettability.

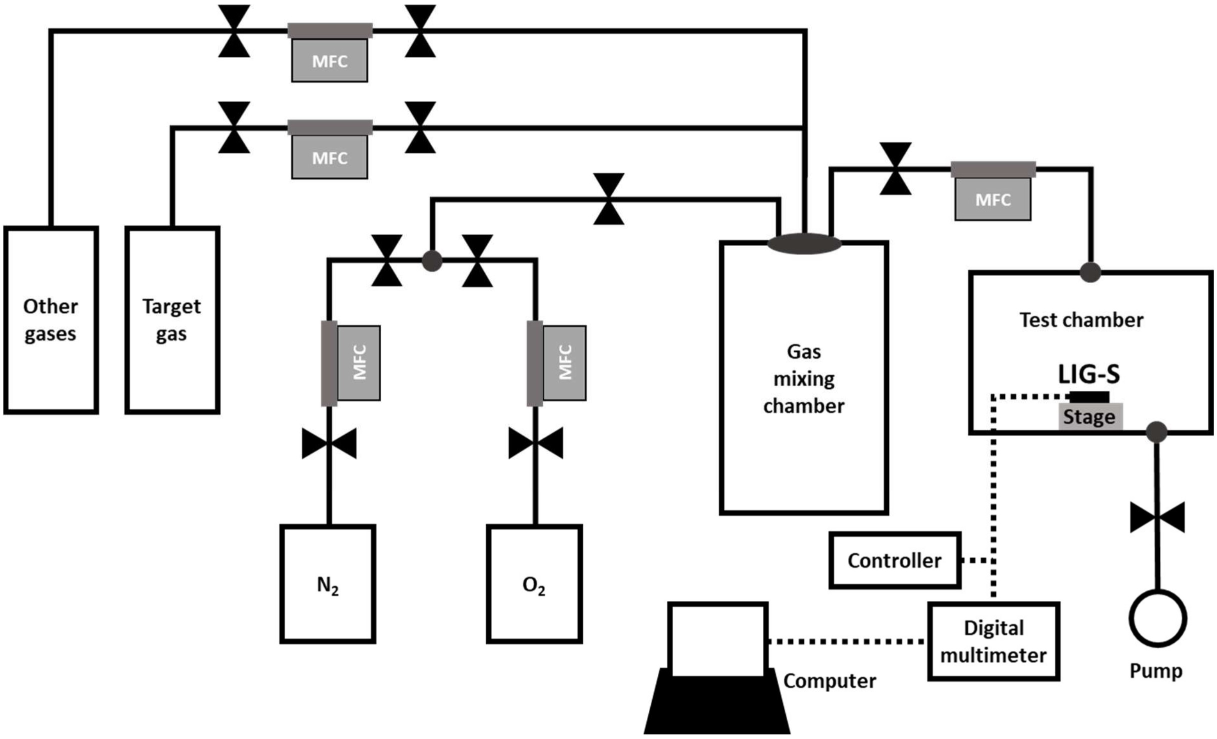

Figure S10. Illustration of the setup for measuring gas-sensing performance of the LIG-S electrodes.

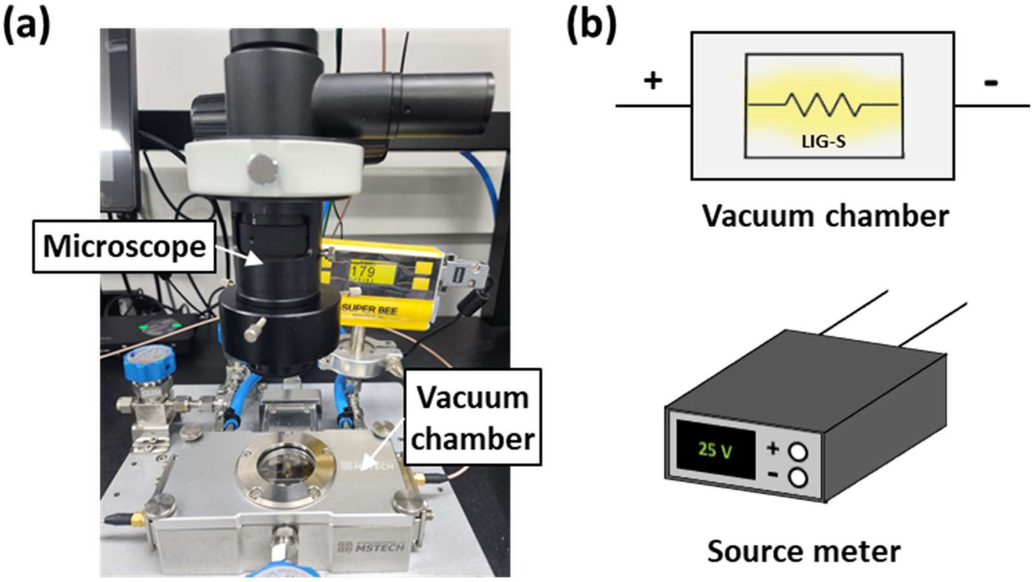

Figure S11. The detailed experimental configuration for resistive heating of LIG-S filament. (a) Optical setting for capturing luminescence changes in response to applied voltage. (b) Schematic illustration of stepwise resistive heating experimental setup.

Supporting Table

Table S1. Summary of the selected publications on free-standing LIGs

Year	Author	Laser	Process	Precursor form	Shape	Spring constant	Modulus	Strength	Strain	Electrical conductivity
**2018**	Luong et al.[1]	IR, pulse	Laminated object manufacturing (LOM)	Film	Foam	-	~300kPa	~80kPa	-35%	~27.5 S/m
**2022**	Liu et al.[2]	IR, pulse	LIG based addictive manufacturing	Powder	Foam	-	-	~79kPa	±0.5%	~48.1 S/m
**2022**	Yu et al.[3]	IR, pulse	Selective laser transforming	Liquid	Foam	-	~31.8MPa	~4.4MPa	-16%	~4380 S/m
**2020**	He et al.[4]	IR, pulse	Line-by-line laser vertical sweeping	Fiber	Fiber	-	-	~250MPa	+7%	~563.13 S/m
**This work**		532 nm, CW	Pyrolytic jetting	Film	Spring	0.8 ~2.4 N/m	~0.3GPa	-	±40%	~1340 S/m

References

[1] D. X. Luong, A. K. Subramanian, G. A. L. Silva, J. Yoon, S. Cofer, K. Yang, P. S. Owuor, T. Wang, Z. Wang, J. Lou, *Advanced Materials* 2018, 30, 1707416.

[2] F. Liu, Y. Gao, G. Wang, D. Wang, Y. Wang, M. He, X. Ding, H. Duan, S. Luo, *Advanced Science* 2023, 10, 2204990.

[3] W. Yu, W. Zhao, S. Wang, Q. Chen, X. Liu, *Advanced Materials* 2022, 2209545.

[4] M. He, Y. Wang, S. Wang, S. Luo, *Carbon* 2020, 168, 308.
